# Longitudinal urinary biomarkers of immunological activation in covid-19 patients without clinically apparent kidney disease versus acute and chronic failure

**DOI:** 10.1038/s41598-021-99102-5

**Published:** 2021-10-04

**Authors:** Krzysztof Laudanski, Tony Okeke, Jihane Hajj, Kumal Siddiq, Daniel J. Rader, Junnan Wu, Katalin Susztak

**Affiliations:** 1 Department of Anesthesiology and Critical Care, The University of Pennsylvania, Philadelphia, PA USA; 2 Leonard Davis Institute for Healthcare Economics, The University of Pennsylvania, Philadelphia, PA USA; 3 School of Biomedical Engineering, Science and Health Systems, Drexel University, Philadelphia, PA USA; 4 School of Nursing, Widener University, Philadelphia, PA USA; 5 College of Arts and Sciences, Drexel University, Philadelphia, PA USA; 6 Department of Genetics, Perelman School of Medicine, The University of Pennsylvania, Philadelphia, PA USA; 7 Division of Renal Electrolyte and Hypertension, Department of Medicine, The University of Pennsylvania, Philadelphia, PA USA

**Keywords:** Immunology, Nephrology, Acute kidney injury, Chronic kidney disease

## Abstract

Kidney function is affected in COVID-19, while kidney itself modulates the immune response. Here, hypothesize if COVID-19 urine biomarkers level can assess immune activation vs. clinical trajectory. Considering the kidney’s critical role in modulating the immune response, we sought to analyze activation markers in patients with pre-existing dysfunction. This was a cross-sectional study of 68 patients. Blood and urine were collected within 48 h of hospital admission (H1), followed by 96 h (H2), seven days (H3), and up to 25 days (H4) from admission. Serum level ferritin, procalcitonin, IL-6 assessed immune activation overall, while the response to viral burden was gauged with serum level of spike protein and αspike IgM and IgG. 39 markers correlated highly between urine and blood. Age and race, and to a lesser extend gender, differentiated several urine markers. The burden of pre-existing conditions correlated with urine DCN, CAIX and PTN, but inversely with IL-5 or MCP-4. Higher urinary IL-12 and lower CAIX, CCL23, IL-15, IL-18, MCP-1, MCP-3, MUC-16, PD-L1, TNFRS12A, and TNFRS21 signified non-survivors. APACHE correlated with urine TNFRS12, PGF, CAIX, DCN, CXCL6, and EGF. Admission urine LAG-3 and IL-2 predicted death. Pre-existing kidney disease had a unique pattern of urinary inflammatory markers. Acute kidney injury was associated, and to a certain degree, predicted by IFNg, TWEAK, MMP7, and MUC-16. Remdesavir had a more profound effect on the urine biomarkers than steroids. Urinary biomarkers correlated with clinical status, kidney function, markers of the immune system activation, and probability of demise in COVID-19.

## Introduction

Individuals exposed to the SARS-Cov-2 virus may develop a spectrum of clinical presentations including but not limited to acute kidney injury (AKI)^1–3^. High variability of clinical presentation is believed to be secondary to the individual inflammatory response and other host-specific traits, particularly in chronic and acute kidney injury^1–7^. Given the unpredictability of the immune response, it is critical to understand the nature of the immune system status in response to critical illness in general and COVID-19 specifically^8,9^. However, predicting the inflammatory response is complex and often require intervention with blood collection, which is burdensome. Prior sepsis studies supported the use of urine samples as biomarkers for therapeutic and immunological monitoring^10–17^. Therefore, collecting urine samples for immune system monitoring may be an appealing alternative over blood sample collection, especially considering that many patients with COVID-19 infections receive treatment at home and blood draw can be challanging^18,19^. While the literature indicates a correlation between these two compartments, it is not a uniform finding across the different research studies and are mainly focused on nucleic acid signatures^19,20^. Also, the link between patterns of immune system activation in urine to COVID-19 outcomes remains unknown.

The kidney is an essential immunological organ participating in several pivotal aspects of the immune system activation, including cytokines (CXCL13, monocyte chemoattractant protein) and several inflammatory mediators processing^21–24^. Kidney dendritic cells are critical to shaping immune response as due to their ability to sense through pathogens and immunoglobulins generated in response to an inflammatory trigger^23,25,26^. More importantly, the kidney plays a critical role in suppressing the response to autoantigens. This aspect may be crucial in COVID-19 as several theories suggest that circulating immunoglobulins lead to vasculitis or other types III immunological hypersensitivity underpinning clinical demise^27,28^. So, whether the kidneys impaired excretory function is related to aberrant immune system response affecting recovery and demise of the critical care illness-affected individuals is an interesting question. Outside COVID-19, loss of the kidney's regulatory process is linked to persistent inflammation, especially long-term, demonstrated by progressive health impairment in patients with end-stage renal disease^22^. This loss of immunological function is distinct from excretory and electrolyte homeostatic purpose of kidney.

The effect of the cytokine storm components on the potential initiation, acceleration and persistence of renal failure remains to be charted in COVID-19 patients. MCP-1 and IL-18 were shown to play a role in progressive kidney failure during recovery from CCI^29–34^. So, the composition of the urine inflammatory markers during cytokine storm may put certain patients at higher risk of incomplete kidney recovery after acute COVID-19 recovery, especially if inflammation persisted. Some of these markers have critical functions in response to viral infections^35–37^. On the other hand, several medications were tried in patients with COVID-19 acute infection. Their mechanisms of action and span from modulating immune system response to inhibit viral replication are different^38–40^. However, their impact on immune response in COVID-19 is just being recognized, but it is exploratory at best^41–44^.

Conversely, the immune system's activation is believed to be the predominant driver of recovery or demise on systemic and local kidney inflammation level^21,45–48^. The kidney function can be further impaired by non-directly driven immunological abnormalities of CCI, leading to tissue hypoxia and function impairment ^9,46,49,50^. The latter process can be fueled by other components of multiorgan failure, suggesting that a specific pattern of biomarkers in the urine may reflect specific organ impairment. Furthermore, there is increased appreciation that kidney- or systemic-mediated recovery patterns can be measured in blood, providing predictive information about COVID-19 and potentially affected outcomes and treatment.

This research study aimed to establish the role of critical mediators during immune system activity using targeted proteomics analysis of urine as the alternative to blood biomarkers. First, we sought to explore the targets for immune system activation by using longitudinal observation after the onset of COVID-19. Second, we sought to determine the effect of demographic variables, preexisting comorbidities, clinical status, various organ dysfunction, and COVID-19-specific treatment on urine markers. Finally, the difference in the levels of urine and blood markers of immunological activation were compared in patients without kidney impairment, patients with pre-existing chronic renal failure, and acute kidney injury.

## Materials and methods

The Institutional Review Board at the University of Pennsylvania approved the study (#813913). The study was performed according to the ethical guidelines of the 2003 Helsinki Declaration. Written informed consent was obtained from all enrolled patients.

### Study populations

Patients admitted to the University of Pennsylvania Hospital system with positive COVID-19 test by polymerase chain reaction were recruited for the study during admission to the hospital. Patients who were pregnant or younger than 18 years of age were excluded from the study.

Clinical data on the enrolled participants were collected from the electronic medical records. Demographic data were collected using preexisting data and were self-determined by the patients. The history of preexisting chronic kidney disease was elucidated from notes in the medical records. Organ failures were characterized using a framework from GlueGrant by manual extraction of medical team notes at the time of blood draw^51^. Respiratory failure (Rf) was defined as using biphasic non-invasive ventilation, intubation, or ECMO engagement. Cardiovascular failure (CVS_f_) was synonymous with shock requiring vasopressor medications secondary to hypotension or heart failure. Renal failure (AKI_f_) was determined by serum creatinine rise criteria consistent with a RIFLE criterion in terms of creatinine increase^52^. Central nervous system failure (CNS_f_) was defined as stroke, seizure or delirium during the hospitalization. Liver failure (L_f_) was defined as an elevation in alanine aminotransferase or asparagine transferase, total bilirubin, or ammonia above the hospital reference value^51^.

We extracted utilization of remdesivir and convalescent plasma, which were highly protocolized per hospital policy according to the FDA recommendations for COVID-19 treatment. Steroid treatment was determined as applying steroid-like compounds to treat COVID-19 pneumonia per healthcare provider notes.

The burden of chronic disease was calculated as Charlson Co-morbidity Index (CCI)^53^. The Acute Physiology And Chronic Health Evaluation III (APACHE III) was calculated within one hour (APACHE1hr) and at 24 h after admission (APACHE24hrs)^54^. The severity of the overall condition was determined by Marshalls Organ Dysfunction Score (MODS) and Sequential Organ Failure Assessment (SOFA)^55^. The survival was determined at 28 days.

A total of 68 patients were enrolled in the study. Their clinical characteristics are presented in Table 1.

**Table 1:** Demographical and clinical characteristic of the studied sample.

Demographics (68 Patients)					
Age [X ± SD]		61.13 ± 18.13			
Age	Below 60 [%]	41.20%			
	Over 60 [%]	58.80%			
Gender	Male [%]	57.35%			
	Female [%]	42.65%			
	Not reported [%]	0.00%			
Race	Caucasian/Hispanic Latino [%]	22.06%			
	Black [%]	66.18%			
	Other/Asian/Unknown [%]	11.76%			
Pre-Existing Conditions					
Charleston Comorbidity Index [X ± SD]		3.82 ± 2.94			
MI [%]		2.94%			
CHF [%]		13.24%			
PVD [%]		5.88%			
CVA/TIA [%]		11.76%			
Dementia [%]		5.88%			
COPD [%]		13.24%			
CTD [%]		4.41%			
Peptic Ulcer Disease [%]		2.94%			
Liver Disease [%]		1.47%			
DM [%]		38.24%			
Hemiplegia [%]		5.88%			
CKD [%]		26.47%			
Solid Tumour [%]		10.29%			
Leukomia [%]		1.47%			
Lymphoma [%]		0.00%			
AIDS [%]		1.47%			
Smoker	Smoker [%]	7.35%			
	Former Smoker [%]	33.82%			
	Non-Smoker [%]	58.82%			
Vaper [%]		0.00%			
Clinical Characteristics					
Mortality [%]		20.59%			
Length of Stay [X ± SD]		22.44 ± 30.1			
ICU [%]		60.29%			
Intubated [%]		42.65%			
ECMO [%]		13.24%			
APACHE Admission + 1Hr [X ± SD]		12 ± 8.44			
APACHE Admission + 24Hrs [X ± SD]		12.06 ± 7.7			
Organ Failures					
		H1	H2	H3	H4
CNSf [%]		13.24%	7.35%	11.76%	8.82%
CVf [%]		38.24%	19.12%	26.47%	16.18%
Rf [%]		41.18%	45.59%	38.24%	20.59%
Rf (AKIf) [%]		30.88%	19.12%	22.06%	11.76%
Lf [%]		55.88%	36.76%	32.35%	16.18%
Bf [%]		47.06%	42.65%	32.35%	22.06%

### Sample collection

After obtaining informed consent, blood and urine samples were collected on four subsequent occasions if possible: within 24 h after admission, followed by 48 h, seven days, after seven days. To account for different timing, we adjust the time designation of all samples based on the date when a patient was admitted to the hospital, dividing samples as collected within 48 h after admission (H_1_), between 49 and 96 h after admission (H_2_), between 3 and 7 days since admission(H_3_), and above seven days (H_4_) (Supplemental Fig. 1). All samples H_1_-H_3_ were collected during patient hospitalization. Long-term H_4_ samples were collected during routine visits to the hospital after discharge.

**Figure 1:**
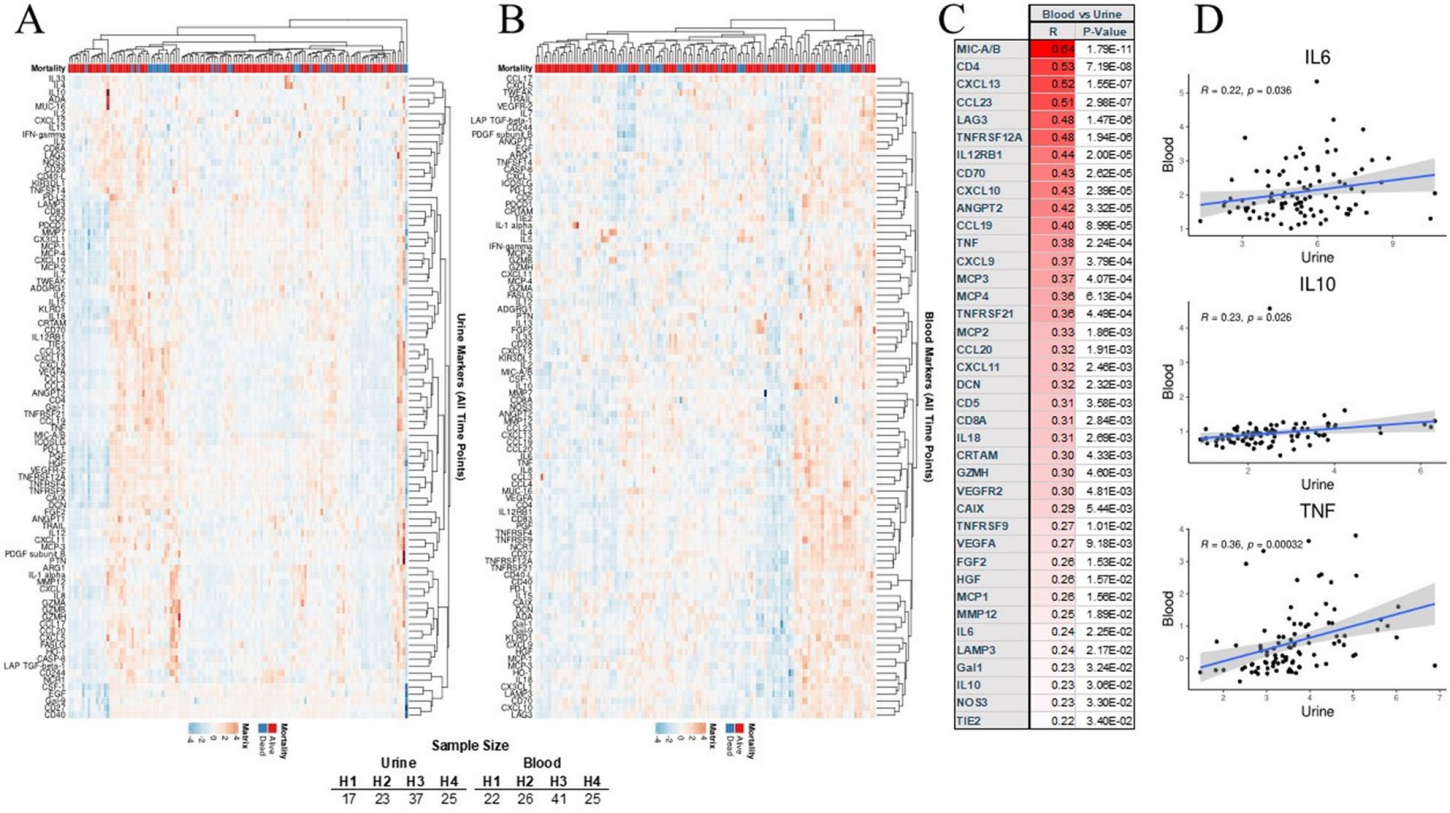
Urine (**A**) and blood (**B**) had distinctive biomarkers with several correlations between urine and blood markers (**C**), with IL-6. IL-10 and TNFα (**D**) in COVID-19 patients.

Blood was collected in heparin vacutainer tubes (BD, Franklin Lakes, NJ) and put on ice. Serum was separated by collecting the top layer after spinning the line at 1000×*g*, 10 min, 4 °C within 3 h from collection. Aliquot serum was stored at − 80 °C. Before shipment, the serum was inactivated by incubation of 100 µl of serum with 5% Tween-20 (Bioworld, Baltimore, MD) for 20 min at room temperature and shipped for analysis or locally tested with ELISA (BioLegend, San Diego, CA).

Urine was collected using standardized equipment, aliquoted, and stored at − 80 °C.

### Serum analysis

The commercial supplier employed O-link technology to assess the serum level of immunological markers. We targeted biomarkers based on the preliminary data and literature review (Supplemental Material #2)^7,49,56–60^. Triton X-100 inactivated blood (n = 102) and urine (n = 114) samples collected from patients with COVID-19 were analyzed using an Olink panel (OLINK Bioscience, Uppsala, Sweden) (Supplementary Fig. 1). The kit provides a microtiter plate for measuring 90 proteins, with data presented as Normalized Protein Expression (NPX) values plotted against protein concentration (pg/mL)^61^. The obtained results are presented as dimensionless values allowing for comparison of the measured protein across different variables^61^. Unfortunately, Olink technology does not allow for relating different cytokines to each other^61^.

Proteins of interest were measured with enzyme-linked immunoassays (ELISA) of interest (Biolegend, San Diego CA). S-spike protein, IgG, and IgM were measured using commercially available kits (RayBiotech, Peachtree Corners, GA). IL-6 was measured using a multiplex kit (Theromofisher, Waltham, MA) on a MagPix machine (Luminex; Austin, TX).

### Statistical analysis

All samples were used for analysis as described in Supplementary Fig. 1 unless stated otherwise. The Lavene test and distribution plots were used to test the normality of variables. Parametric variables will be expressed as mean ± SD and compared using *t*-Student. Multiple groups were compared using ANOVA with subsequent *t*-Student tests for intra-group comparisons. The NPX values for urine and blood were bridge normalized using the *OlinkAnalyze* R package^62^. For non-parametric variables, median (Me) and interquartile ranges (IR) will be shown with U-Mann–Whitney statistics employed to compare such variables. The correlation was calculated as *r*^2^-Pearson momentum. A double-sided *p *value less than 0.05 will be considered statistically significant for all tests, but we took a more conservative value 0.01 or less in most of the analyses. Statistical analyses will be performed with Statistica 11.0 (StatSoft Inc., Tulsa, OK), the R programming language, and SPSS v26 (IBM, Endicott, NY).

### Ethics approval and consent to participate

The Institutional Review Board approved the study at the University of Pennsylvania (#813913).

### Consent for publication

All authors reviewed the manuscript and consented to publication.

### Author’s statement

All authors reviewed the final version of the manuscript and agreed to its publication.

## Results

### Patterns of urine markers of activation and relationship to immunological activation and s-protein levels

**Figure 2:**
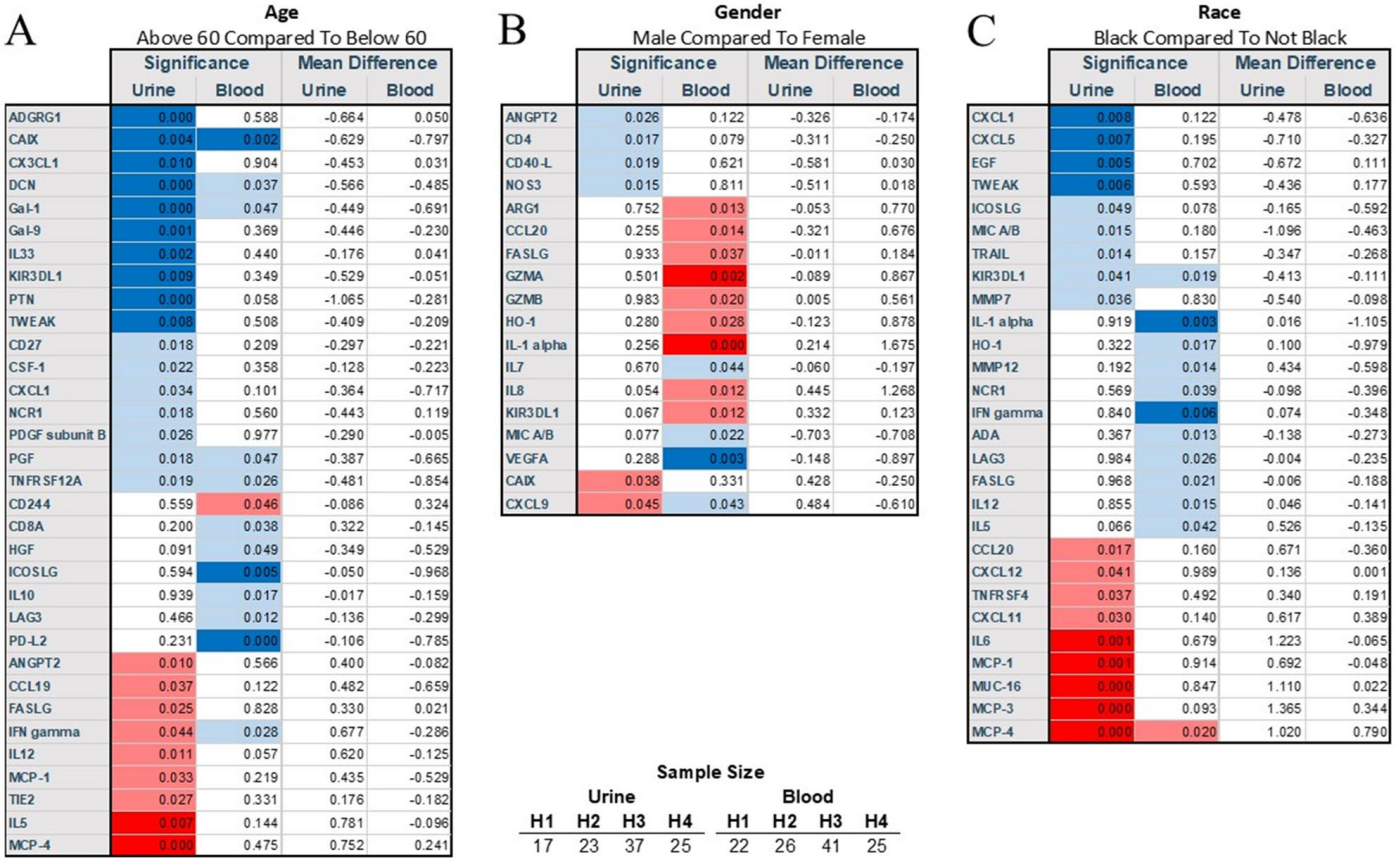
Demographic variables include age (**A**), gender (**B**), and self-declared race (**C**) affected the immunological profile in COVID-19 patients.

Urinary biomarkers demonstrated a unique pattern in study samples that were somewhat different from blood samples (Fig. 1A,B; Supplemnetary Fig. 2A,B). Several biomarkers were elevated while others were low, and one with high variability, but the overlap between blood and urine was uneven. Their urine levels were quite dynamics over the course of disease (Supplementary Fig. 3).

The correlation between urine and blood markers was strong for 37 out of 90 (Fig. 1C; Supplemental Fig. 2A-C; Supplemental Fig. 3). Most importantly, IL-6, TNFα, and IL-10 showed a significant correlation between urine and serum samples (Fig. 1D). In addition, correlations between these markers in urine and blood were statistically significant, suggesting that urine can be used to sample the patients’ immune system (Supplement Fig. 1B). Finally, IL-6 levels in urine measured with O-link technology followed a similar trend as using more conventional technology (Supplemental Fig. 2C). Several other markers had similar strong correlations between urine and serum (Supplemental Fig. 2D).

**Figure 3:**
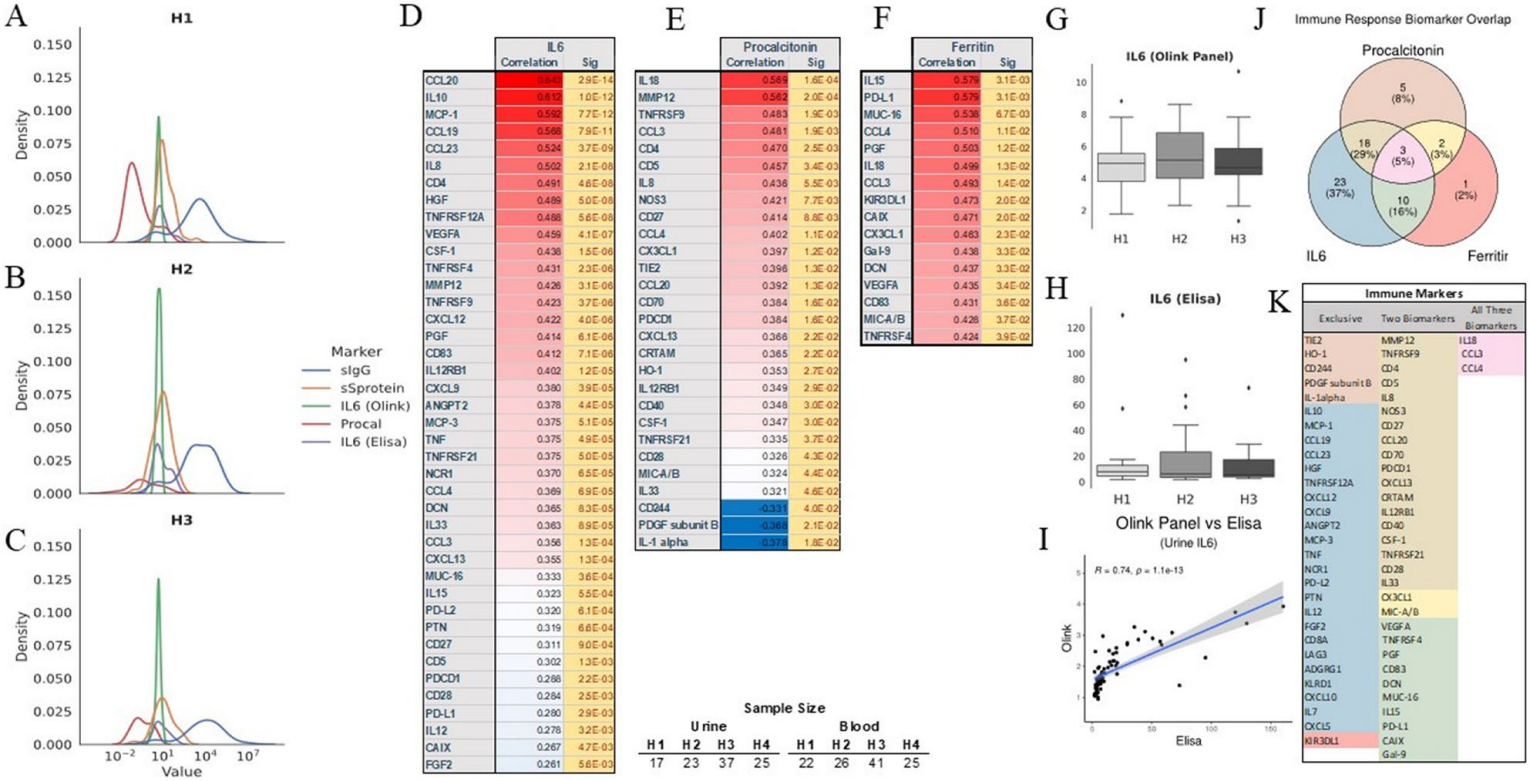
Serum spike protein, IgG, and procalcitonin followed a typical course of the disease over H1 (**A**), H2 (**B**), H3 (**C**) period. Urine IL-6 (**D**) correlated with several urine immunological markers, while procalcitonin correlations were much less abundant (**E**) and correlation with ferritin relatively sparse (**F**). IL-6 measured via O-link (**G**) and ELISA (**H**) followed expected clinical trajectory and correlated highly with each other (**I**). Several inflammatory markers correlated with Olink value (**J**) but very heterogeneously and marker dependent (**K**).

### Demographical and preexisting clinical patients’ characteristics and their effect on urine immunological markers

Older patients had less pronounced levels of CAIX (Fig. 2A). ADRG1, CXCL1, DCN, Gal-1, Gal-9, IL-33, KIRDL1, PTN, and TWEAK, while MCP-4, IL-5, were more often present more in the urine of older patients (Fig. 2A). Very few cytokines showed gender-specific differences, but in male individuals, GZMA and IL-1α were higher, while VEGFA was lower. (Fig. 2B). Black subjects had a higher level in IL-6, MCP-1, MCP-3, MCP-4, and MUC-16, but TWEAK, CXCL1, CXCL5, and EGF were depressed (Fig. 2C). These differences were not reflected in the blood samples. We also observed differences in serum levels of IL-1α and IFNγ in African American samples (data not shown).

The burden of pre-existing condition correlated very strongly with urine level of DCN (*r*^2^ = 0.55; *p* = 3.41 × 10^–10^), CAIX (*r*^2^ = 0.43; *p* = 1.950 × 10^–6^) and PTN (*r*^2^ = 0.43; *p* = 3.323 × 10^–6^), but inversely with IL-5 (*r*^2^ = − 0.3; *p* = 1.498 × 10^–3^) or MCP-4 (*r*^2^ = 0.3; *p* = 1.553 × 10^–3^) most significantly (Supplemental Fig. 4A).

**Figure 4:**
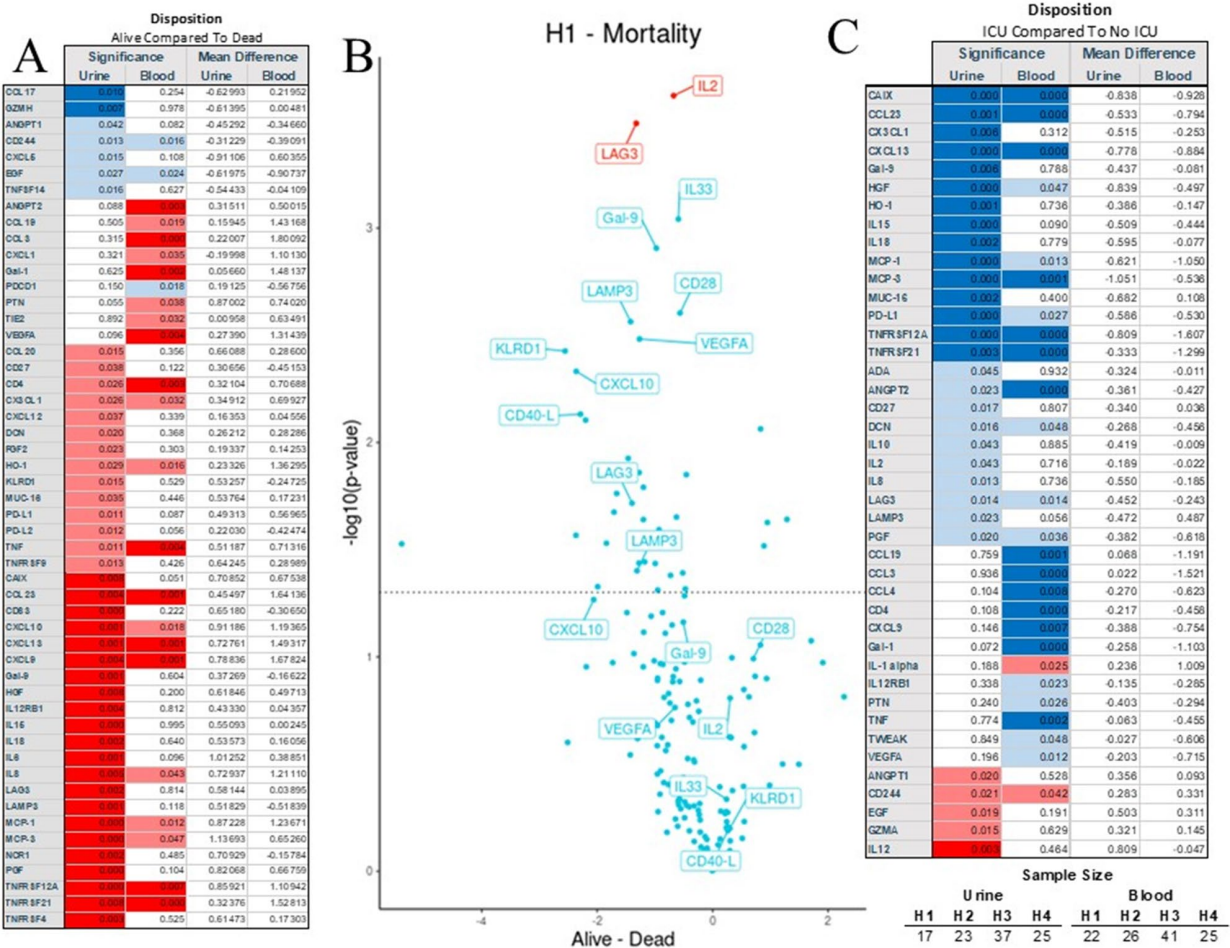
Several markers were elevated in demised patients (**A**), but IL-2, and LAG-3 were the only predictor of demise at admission (**B**). Admission to the ICU was demonstrated by downregulation of several markers with only one significantly elevated (**C**).

### The immune response and urine biomarkers

Serum spike protein and IgG followed the typical pathogen and exposure–response (Fig. 3A&B&C). In addition, the inflammatory markers followed the expected trajectory with an initial increase in procalcitonin and ferritin followed by normalization (Fig. 3A&B&C).

The serum level of spike protein correlated with CCL20 (*r*^2^ = 0.29; *p* = 0.03), TWEAK (*r*^2^ = 0.23; *p* = 0.016), and GZMA (*r*^2^ = 0.21; *p* = 0.028) exclusively. The serum level of IgG correlated positively with PDCD1 (*r*^2^ = 0.2; *p* = 0.041) while negative with MCP-1 (*r*^2^ =  − 0.2; *p* = 0.044), GZMB (*r*^2^ = 0.21; *p* = 0.034), and IFNγ (*r*^2^ = 0.22; *p* = 0.03). Blood IL-6 level correlated with several urine markers (Fig. 3D), but those correlations were much less frequent for procalcitonin (Fig. 3E) or ferritin (Fig. 3F). IL-6 in urine and blood normalized eventually after the initial increase (Fig. 3G & H).

The normalized level of IL-6 measured as NPX vs. absolute value correlated highly (*r*^2^ = 0.74, *p* = 1.11 × 10^–13^) (Fig. 3J). In addition, there was a significant overlap between significant urine markers and all three different markers of inflammation, with IL-18, CCL3, and CCL4 were the commonest markers (Fig. 3K&L).

### Disease severity and urine biomarker patterns

We identified several biomarkers that significantly changed with respect to mortality. LAG-3 and IL-2 shown the most differences in the initial samples (Fig. 4A&B). Length of stay in the hospital correlated significantly with several markers, but most significantly with MCP-3 (*r*^2^ = 0.37; *p* = 8.032 × 10^–5^), MUC-16 (*r*^2^ = 0.37; *p* = 3.27 × 10^–4^), EGF (*r*^2^ = − 0.39; *p* = 3.994 × 10^–5^) and CXCL5 (*r*^2^ = − 0.32; *p* = 6.499 × 10^–4^) (Supplemental Fig. 4B). The patients admitted to the ICU demonstrated increased IL-12 while CAIX, CCL23, Gal-9, HGF, HO-1, IL-15, IL-18, MCP-1, MCP-3, MUC-16, PD-L1, TNFRS12a, and TNFRS21 were depressed (Fig. 4C). The admission and 24 h APACHE analysis demonstrated positive correlations with TNFRS12 (*r*^2^ = 0.654, *p* = 6.84 × 10^–15^), PGF (*r*^2^ = 0.565, *p* = 1.05 × 10^–10^), CAIX (*r*^2^ = 0.516, *p* = 8.10 × 10^–10^), and DCN (*r*^2^ = 0.502, *p* = 2.06 × 10^–8^), while CXCL5 (*r*^2^ = − 0.289, *p* = 0.002) and EGF (*r*^2^ = − 0.208, *p* = 0.068) were negatively correlated (Supplemental Fig. 4C). After removing patients without AKI, several markers persisted (Supplemental Fig. 5A &B).

**Figure 5:**
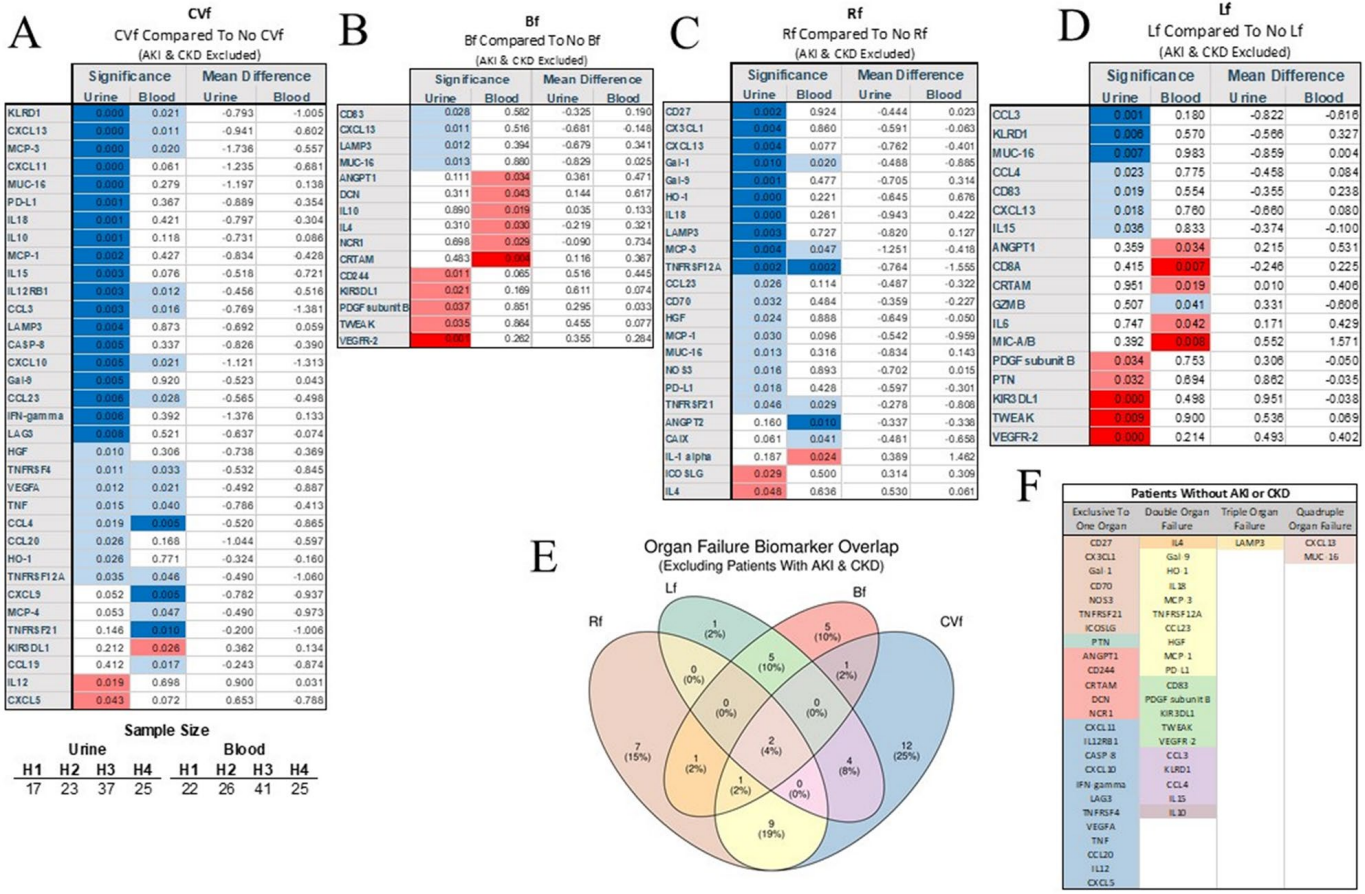
Cardiovascular failure (**A**), utilization of pressors (**B**), respiratory failure (**C**), and liver failure (**D**) produced specific patterns of the injury. Several markers seem to be specific for organ failures (**E** & **F**).

### Urine biomarkers pattern of activation specific for organ failures

We found that patients requiring hemodynamic support due to the vasoplegia of heart failure during COVID-19 demonstrated depressed levels of KLRD1, CXCL13, MCP-3, CXCL11, MUC-16, PD-1L, IL-18, IL-10, MCP-1, IL-16, IL-12RB1, CCL3, LAMP3, CASP8, CXCL10, Gal-9, and CCL-23 in urine (Fig. 5A). Serum lactic acid correlated weakly with urine markers of CASP-8 (*r*^2^ = 0.28; *p* = 0.004), PCDC (*r*^2^ = 0.22; *p* = 0.024), IL-12 (*r*^2^ = 0.21; *p* = 0.031) and ICOSLG (*r*^2^ = 0.2; *p* = 0.05). Patients on pressor had elevated urine markers: CCL20, CCL23, HGF, IL-15, IL-2, TNFRSF12A (data not shown). Patient with abnormalities of central nervous had elevated levels of serum VEGF* r*^2^. (Fig. 5B). Patients requiring respiratory failure showed depressed CD27, CXCL1, CXCL13, Gal-1, Gal-9, HO-1, IL-18, LAMP3, MCP-3, TNFRS12A (Fig. 5C). Several elevations signified an emergence of liver failure in the urine level of CCL3, KLRD1, MUC-16, KIRLD1, TWEAK, VEGF* r*^2^ (Fig. 5D). None of these markers were significant altered in blood in patients with liver failure.

Several markers demonstrated organ specificity, but MUC-16 and CXCL13 were uniformly elevated in all cases of organ failure, while several others were specific for a particular organ failure (Fig. 5E&F).

### The immunological profile of patients with preexisting or newly emerged renal failure

**Figure 6:**
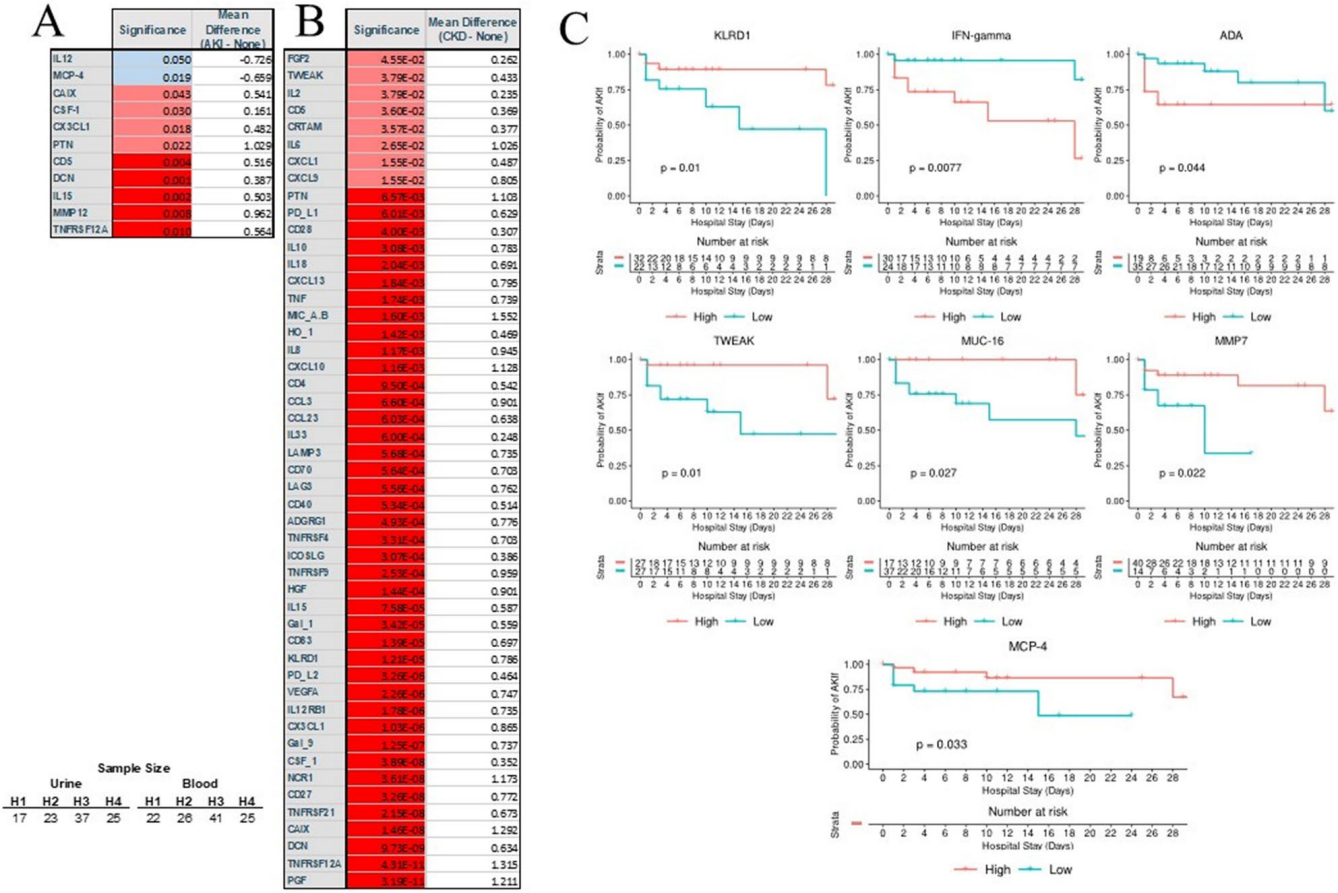
AKI was signified by several markers of injury in urine (**A**) but preexisting renal disease induced much more significant changes in urine markers (**B**). Several markers were good predictors of the emergence of AKI over the course of COVID-19 (**C**).

We identified patterns among patients with newly acquired acute kidney failure versus patients with preexisting chronic kidney failure compared to individuals without acute or chronic kidney failure (Fig. 6A). Out of 31 cases with AKI, patients who recovered (n = 20) had increased urine levels of MCP-1 (*p* = 0.001) and MCP-3 (*p* = 0.008) as compared to those who did not recover (n = 11) (Supplemental Fig. 6A,B). The most striking finding was the observation of massive raise of numerous activation biomarkers in urine seen in patients with preexisting chronic kidney failure (Fig. 6B). Patients with AKI had few markers elevated in urine (CD5, DCN, IL-15, MMP12, TNFRS21). High IFNγ at admission was the only predictor of developing kidney failure (Fig. 6C). Elevated at admission, TWEAK, KRLD1, MMP7, MUC-16, and MCP-4 were predictors of AKI development during a hospital stay.

Out of 31 individuals with AKI, 20 patients recovered while the remaining died. MCP-1 (*p* = 0.001) and MCP-3 (*p* = 0.008) were significantly depressed as compared to non-AKI patients (Supplemental Fig. 5).

### The effect of anti-COVID-19 therapies on immunological profile in the urine

**Figure 7:**
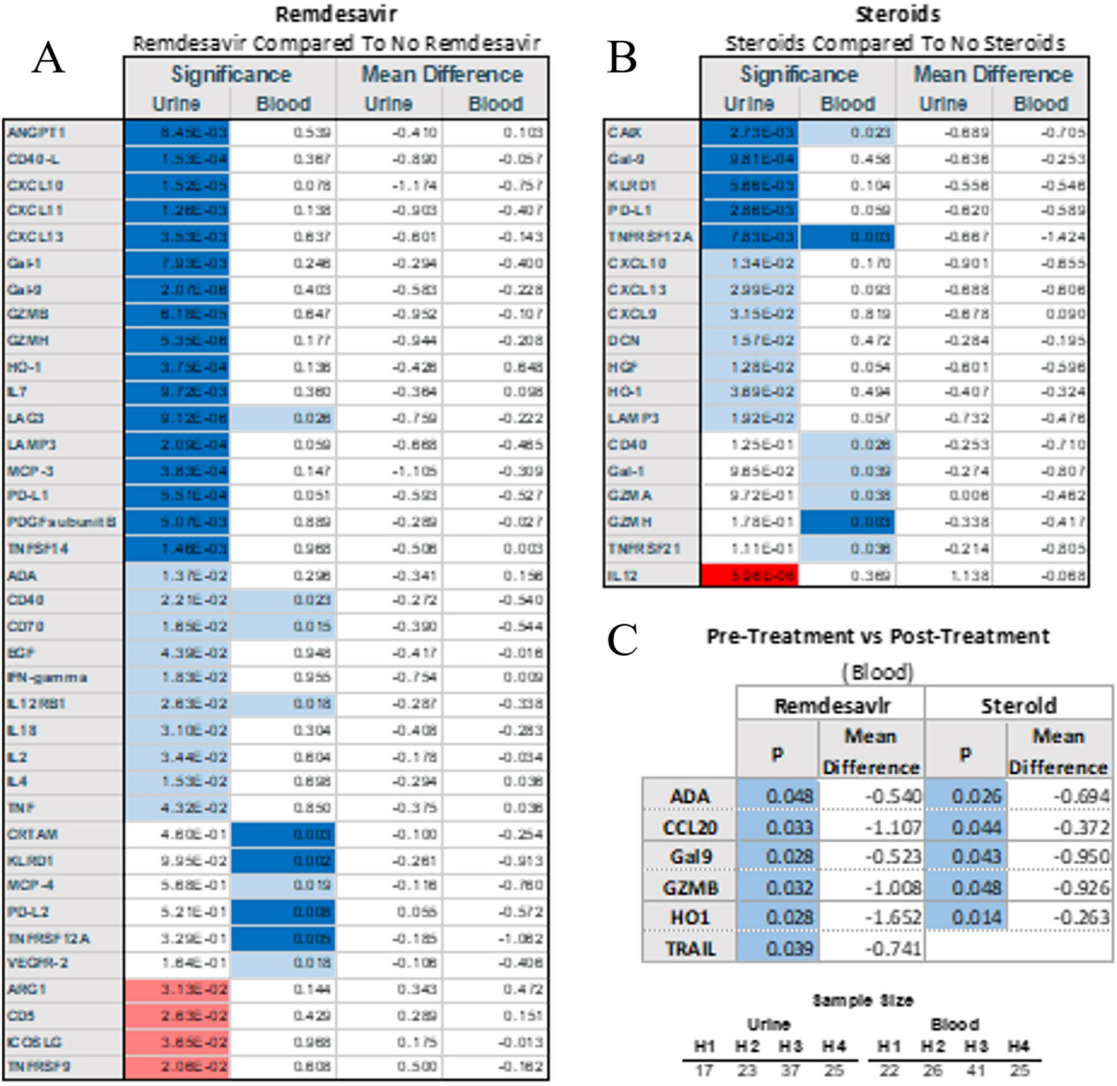
Remdesivir (**A**) was correlating with much more dynamic activation pattern than steroids (**B**) judging from presence of the urinary markers in urine. Only few markers in blood were changed after introduction of remdesivir or steroids (**C**).

The engagement of two major treatment modalities, remdesivir and steroids have a distinctive effect on urine and blood profile (Fig. 7A&B). Higher urine IL-12 was the most prominent feature of steroid therapy. However, several other markers were depressed by remdesivir (ANGPT1, CD40L, CXCL10, CXCL11, CXCL13, Gal-1, Gal-9, GZMB, GZMH, HO-1, IL-7, LAG-3, LAMP3, MCP-3, PD-1L, PDGF subunit B) or steroids (CAIX, Gal-9, KLRD1, PD-1L, TNFRS12A) with Gal-9 and PD-1L overlapping (Fig. 7A&B).

When compared the patients who were enrolled in the treatment longitudinally (pre- *vs* post-treatment) we found that one marker was changed in urine after inititation of remdasavir (CCL4; *p* = 0.047) and none for steroids. When blood was profiled the same way we found six immunological biomarkers in case of blood profiling in patients treated with remdasavir and five with steroids (Fig. 7C).

## Discussion

This is the first manuscript demonstrating targeted immunological profiles in the urine of patients with acute COVID-19 infection. It is also one of few studies demonstrating the utilization of urine as the source of immunological information^11,14,16,19^. The major value of the presented research is a demonstration that several immunological markers correlated with activation of the immune system, the severity of the disease, and clinical outcomes while using urine as the source material. Due to the limitation of Olink technology, the absolute value of the protein is unknown, though we did cross-validate IL-6 level with another technique. Although the level cannot be related to serum concentration due to technological limitations, it provides insight other similar studies where the relative differences were compared, not absolute levels^19,61^.

The use of urine as the source of material to assess the activation of the immune system is infrequent despite its availability. Bandyopadhyay et al*.* utilized urine to detect early sepsis, but they measure gene expression in the cellular fraction of urine^19^. The process of analysis was augmented by artificial intelligence. Here, we focus on protein present in the urine. Both approaches would be complementary as cellular RNA expression denotes the activity of the cells translocated or shed into urine while protein measured in urine creates a screenshot of the inflammatory environment^12,17,19^. Serum IL-6, procalcitonin, and ferritin had several positive correlations between numerous markers, with members of CCL protein being the most common. Elevation in IL-6, IL-15, IL-2, monocyte attractant proteins, and the CXCL family suggest significant activation of the immune system, particularly consistent with the idea of the cytokine storm^3,8,58^. Elevation in receptors for programmed death may be reflective of increased apoptosis seen in patients with sepsis^58^. The source of proteins cannot be ascertained from the existing data sets, but they describe the clinical evolution and immunological response well in COVID-19 or viral infection^7,30^.

Our immunological profiling revealed that urine CCL23, CXCL13, IL-15, CD5, several members of the TNFαR family, and monocyte chemoattractant protein correlated with blood levels. Prior studies indicated that these molecules are an essential component of the immunological response in COVID-19 and other viral infections^3,5,7,8,28,56,60,63^. Though the dynamics of the investigated biomarkers seem to be less exaggerated as compared to blood levels, they were related to increased mortality, organ failure, and unfavorable outcome. Several, but fewer than in blood, markers were more prominently expressed in the urine than blood. This is not surprising since urine is an environment prone to significantly fewer immunologically active cells than blood. Proinflammatory interleukins, monocyte chemoattractant proteins, and TNFα receptor superfamily are the most prominent biomarkers correlating with mortality, length of stay, or APACHE. MUC-16, CCL2, CCL3, CXCL13, EGF, CD40, CD27, CSF-1, and MMP-7 demonstrated consistent elevation across all samples irrespective of the origin. Increased levels of MCP were reported before and linked to unfavorable outcomes secondary to the monocyte activation^2,64^. Similar fluctuation of the cytokines was reported before in blood samples obtained from COVID-19^3,5,35,63–65^. The universal presence of MUC-16 is somewhat puzzling, except that this marker has utility in guiding fluid replacement in heart failure^66,67^. Several of our patients had several pressures and fluid requirements often accompanied by heart failure, leading to congestive heart failure that was potentially responsible for MUC-16 elevation^3,8,60^.

The treatment with remdesevir downregulated several markers in the urine but the size oeffect was depending on the analysis. Depression in the urine markers is most likely due to the direct systemic inhibitory effect of remdesevir as excretion in urine is minimal^40^. Viral load is one of the critical determinants of the immune response, but we did not measure it in our system instead focused on immunoglobulin response^65^. Alternatively, we may observe a bias as remdesavir was initially contraindicated during the use of pressors. That indication was changed^39^. Steroids treatment has a much less significant effect. This is most likely a reflection of the heterogeneous nature of our group when the ICU and ambulatory patients were gathered together in our study. Also, the practice pattern may change as steroids were initially contraindicated to be utilized upon completion of some clinical trials. Steroids demonstrated benefit only in a most severe form of COVID-19 triggered ARDS while their application in sepsis is highly debatable^8,38,60,68,69^.

The kidney is an immunologically active organ^21,22,26^. Several markers were highly elevated in a patient's urine if they have preexisting renal failure, suggesting a much more robust inflammatory response to COVID-19. Considering the pivotal role of the kidney in regulating circulating immunoglobulin, it is not surprising that impaired removal of immunoglobulin results in exaggerated immune response, especially in the case of COVID-19^4,21,47^. This is potentially one explanation for less favorable clinical outcomes among patients with preexisting CKD in COVID-19.

Far fewer predictor markers of AKI were seen, including CD5, DCN, IL-15, MMP12, and TNSFRP12A as we initially expected. Their composition does not provide a coherent interpretation as CD5 is a scavenger receptor, decorin is matrix proteoglycan, IL-15 has a critical role in the viral response, and MMP12 has yet to establish a role in aneurysm TNSFRP12A^9,59^. However, in COVID-19, CD5 was seen as part of disease-specific presentation^63^. Kidney failure can be mediated via IL-15 and MMP12, which are often involved in monocyte response—a critical part of COVID-19 activation^2,64^. However, local inflammation is not the only explanation for kidney failure. The emergence of AKI signifies kidney function impairment due to the toxic, potentially immunological, injury, while the most common is hypoxemic injury secondary to demand/supply oxygen mismatch. This mismatch damages the urine excretory function of the kidney, but it may impair other functions, including immunological, of the kidney. Lack of a robust response may indicate that AKI is more likely to occur to the hypotension and hypoperfusion problems than the direct local response of the immune system to the virus. Elevation in MUC-16 in our population suggests severe cardiovascular impairment and is a sign of congestive heart failure^8,27,66^.

Interestingly, the level of several markers at the admission seems to determine the outcome of AKI, which is consistent with cytokine storm early in COVID-19. IFNγ was the most prominent, which is amongst the most potent activators of the immune response in viral infection and the exuberant response was linked to unfavorable sepsis and COVID-19 outcomes^8,28,58,64^. MCP-4, TWEAK, MUC-16, and MMP7 strongly predict recovery from AKI, suggesting that a certain milieu of the cytokine and inflammatory factors must be maintained to optimize recovery^21^. Some of these proteins determine the function of the monocyte and are linked to kidney recovery^2,32–36,64^. Others correlate with the recovery of secondary organs like cardiovascular resulting potentially in the improvement of kidney perfusion^23,66^. However, their essential role in the recovery of AKI can only be established via experimental studies.

The result of our study has to be taken with some considerations. First, though the kidney is an immunologically active organ, the origin of the biomarkers cannot be established. The observed markers could be found in the urine because of apoptosis due to the apoptosis, secretion or shedding^58^. Alternatively, the presence of the biomarkers in the urine may represent metabolism or active inflammatory process^26,48^. Viral particles and immunological cells are routinely found in the urine and may stimulate the immune system^65^. Consequently, our study does not indicate the potential causality. Some authors augmented their analysis using machine learning techniques, but even intense computational techniques cannot prove causality^19^. We did not adjust for the creatinine clearance, but this technique has several controversies, as the effect on preserved glomerular filtration rate on their urine levels is extremely variable and factor-dependent. First, some recommend correction of the urine markers for the serum creatinine. Here, we decided against this technique as this recommendation is not universally recognized. Secondary, the level of inflammatory mediators in urine may be related to immune response, not a secretory function of the kidney. Alternatively, the measurement of total protein could be conducted and used as the normalization. However, the amount of protein in urine depends on several factors in general, while immune biomarkers have even more confounders. For example, a high protein level may correlate with acute tubular necrosis, yet the level of cytokines may be below detectable level due ot the intense metabolism. ATN emerges as the hypoperfusion or inflammatory event, often both. The variable nature of both components would make standardization using the protein difficult. Our manuscript demonstrates that urine markers can be utilized to monitor the pathogenic and clinical changes in the course of viral disease. However, clinical interpretation of the measured level may be different as compared to blood. Biological markers can be passively diffused into usine, actively secreted bu immune system, released during necrosis and apoptosis processes. These veriaty of variables urges caution when analysis thei biological function as there is a scant data available outside this manuscript. In our case, only 37 out of 90 markers correlated with urine and blood, underscoring that other factors may be at play. Our preliminary analysis of the correlation between serum creatinine and several markers demonstrated a multifactorial and non-linear relationship. Finally, our population represented patients in various severity and stages of the disease, potentially biasing results^3,8,56^. However, patient heterogeneity allowed for several analyses despite the introduction of heterogeneity. To focus on more robust findings, we analyzed markers with a much lower *p *value of 0.01 than the customary 0.05. We also limited the number of the biomarkers studied to the ones we believed are most significant in COVID-19 based on literature review^7,49,56–60^. Finally, we could not account for all variables, including smoking, diabetes, preexisting hypertension, which are critical for the performance of the kidney during the infection, or COVID-19 itself^7,8,43,56^. This study is designed as cross-validation, pilot, hypothesize generating study assessing an ability to use urine markers to gauge the immunological response in COVID-19 patients.

## Conclusions

Urine provides a unique insight into the immunological function of the patients with COVID-19, allowing for correlation with clinical status, markers of the immune system activation, and probability of demise.

## Supplementary Information

Supplementary Information.

## Data Availability

The datasets used and/or analyzed during the current study are available from the corresponding authors on reasonable request.

## Abbreviations

ADA: Adenosine deaminase
AKI: Adenylate kinase isoenzyme 1
AKIf: Acute kidney injury failure
ANGPT1: Angiopoietin-1 receptor
ANGPT2: Angiopoietin-2 receptor
Bf: Blood failure
CCI: Charlson comorbidity index
CAIX: Carbonic anhydrase IX
CCL17: C-C motif chemokine 17
CCL19: C-C motif chemokine 19
CCL20: C-C motif chemokine 20
CCL23: C-C motif chemokine 23
CD244: Natural killer cell receptor 2B4
CD27: CD27 antigen
CD27: Cluster of Differentiation 27
CD28: T-cell-specific surface glycoprotein CD28
CD33: Myeloid cell surface antigen CD33
CD4: T-cell surface glycoprotein CD4
CD70: CD70 antigen
CD8: Cluster of differentiation 8
CD83: CD83 antigen
CD8A: T-cell surface glycoprotein CD8 alpha chain
CKD: Chronic kidney disease
CNSf: Central Nervous System Failure
CVf: Cardiovascular Failure
CX3CL1: Fractalkine
CXCL1: Growth-regulated alpha protein
CXCL13: C-X-C motif chemokine 13
EGF: Pro-epidermal growth factor
FasL: Fas antigen ligand
Gal-1: Galectin-1
Gal-9: Galectin-9
HGF: Hepatocyte growth factor
ICOSLG: ICOS ligand
ICU: Intensive Care Unit
IFN-gamma: Interferon-gamma
IL-6: Interleukin-6
IL-7: Interleukin-7
IL-8: Interleukin-8
IL-10: Interleukin-10
IL-12: Interleukin-12
IL-15: Interleukin-15
IL-18: Interleukin-18
KIR3DL1: Killer cell immunoglobulin-like receptor 3DL1
KLRD1: Natural killer cells antigen CD94
LAG3: Lymphocyte activation gene 3 protein
LAMP3: Lysosome-associated membrane glycoprotein 3
Lf: Lung failure
MCP-1: Monocyte chemotactic protein 1
MCP-2: Monocyte chemotactic protein 2
MCP-3: Monocyte chemotactic protein 3
MCP-4: Monocyte chemotactic protein 4
MIC-A/B: MHC class I polypeptide-related sequence A/B
MUC16: Mucin-16
NOS3: Nitric oxide synthase, endothelial
PD-L1: Programmed cell death 1 ligand 1
PD-L2: Programmed cell death 1 ligand 2
PDCD1: Programmed cell death protein 1
PTN: Pleiotrophin
Rf: Respiratory failure
TNF: Tumor necrosis factor
TNFRSF12A: Tumor necrosis factor receptor superfamily member 12A
TNFRSF21: Tumor necrosis factor receptor superfamily member 21
TNFRSF4: Tumor necrosis factor receptor superfamily member 4
TNFRSF9: Tumor necrosis factor receptor superfamily member 9
TWEAK: Tumor necrosis factor (Ligand) superfamily, member 12
VEGFR-2: Vascular endothelial growth factor receptor 2
VEGFR-1: Vascular endothelial growth factor receptor 1
