# Supplementary material for: Longitudinal urinary biomarkers of immunological activation in covid-19 patients without clinically apparent kidney disease versus acute and chronic failure
Source: Sci Rep. 2021 Oct 4;11:19675. doi: 10.1038/s41598-021-99102-5 (PMC8490434; doi:10.1038/s41598-021-99102-5)
Supplement: Supplementary file 1 — Supplementary Information. [file 41598_2021_99102_MOESM1_ESM.pdf]

Supplemental Figure 1. Time distribution of samples for blood and urine

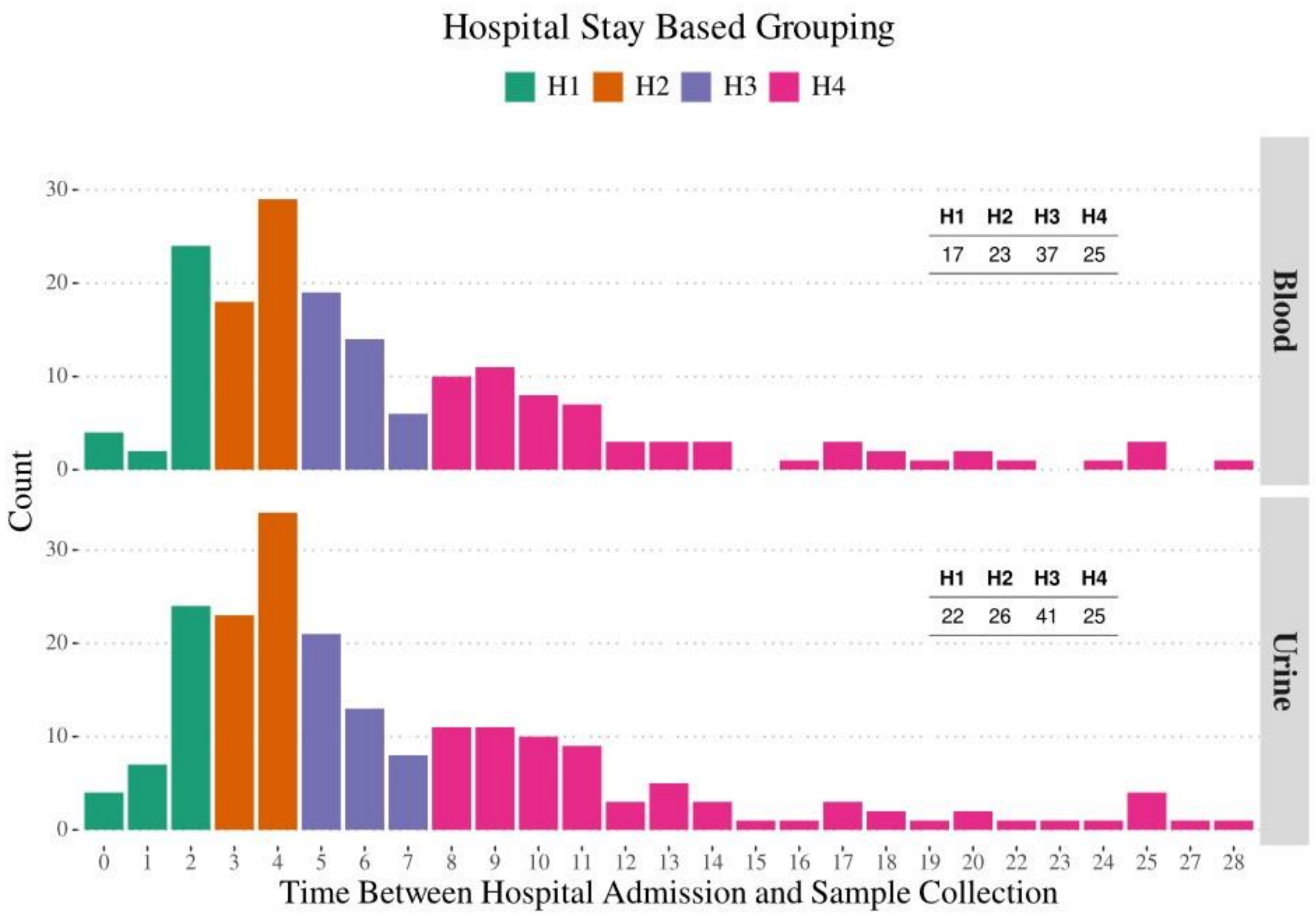


Supplemental Figure 1

Supplemental Figure 2. Correlation between urine and blood markers were significant for several of them (A) and high in general (B). The level of IL-6 in urine measured Olink technique closely followed the level measured with ELISA (C). Several markers had positive correlation between urine and blood (D).

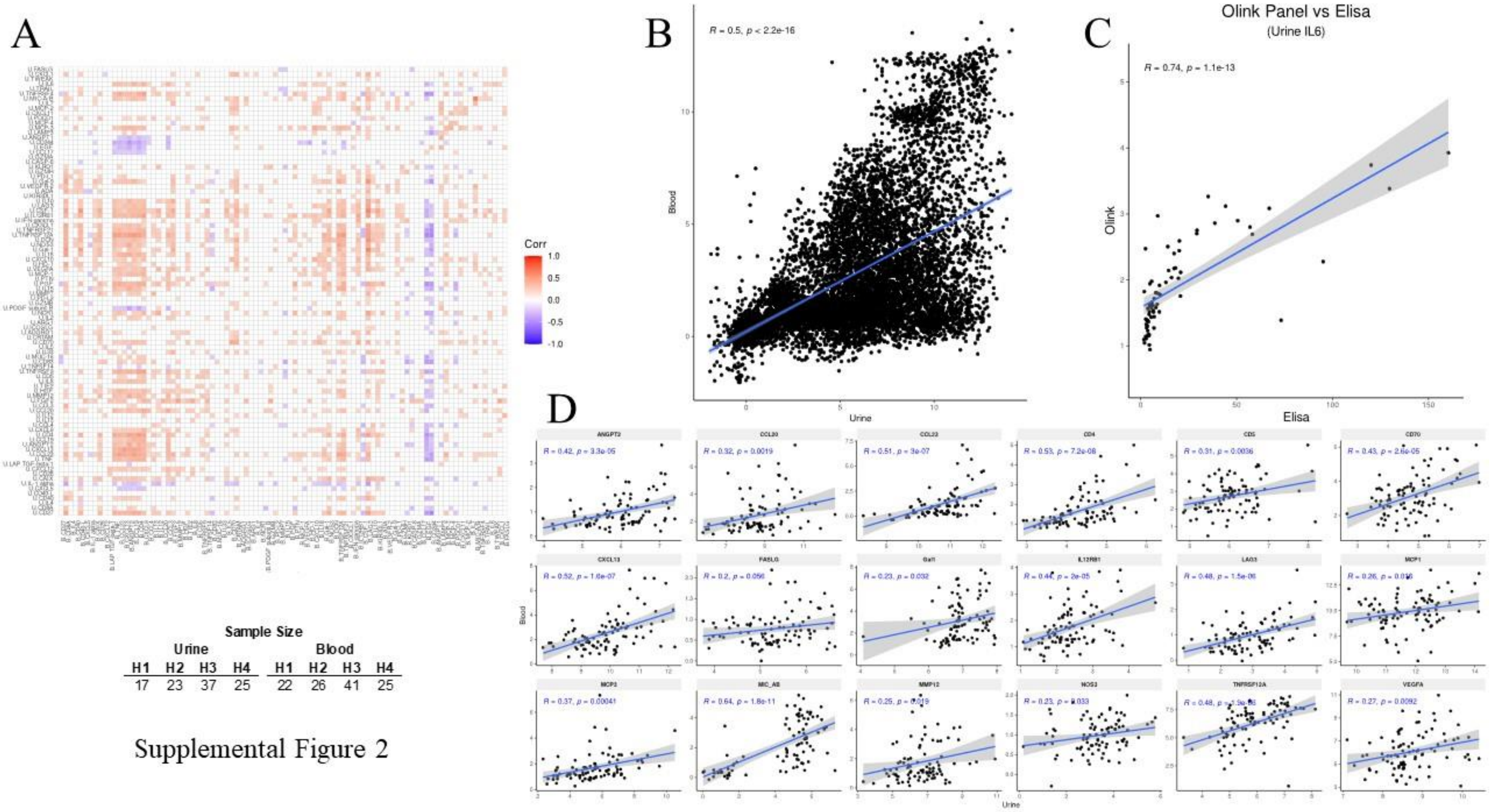


Sample Size

| Urine | | | | Blood | | | |
|---|---|---|---|---|---|---|---|
| H1 | H2 | H3 | H4 | H1 | H2 | H3 | H4 |
| 17 | 23 | 37 | 25 | 22 | 26 | 41 | 25 |

Supplemental Figure 2

Supplemental Figure 3. Dynamic response over time for immunological biomarkers in blood (A) and urine (B) for all studied markers

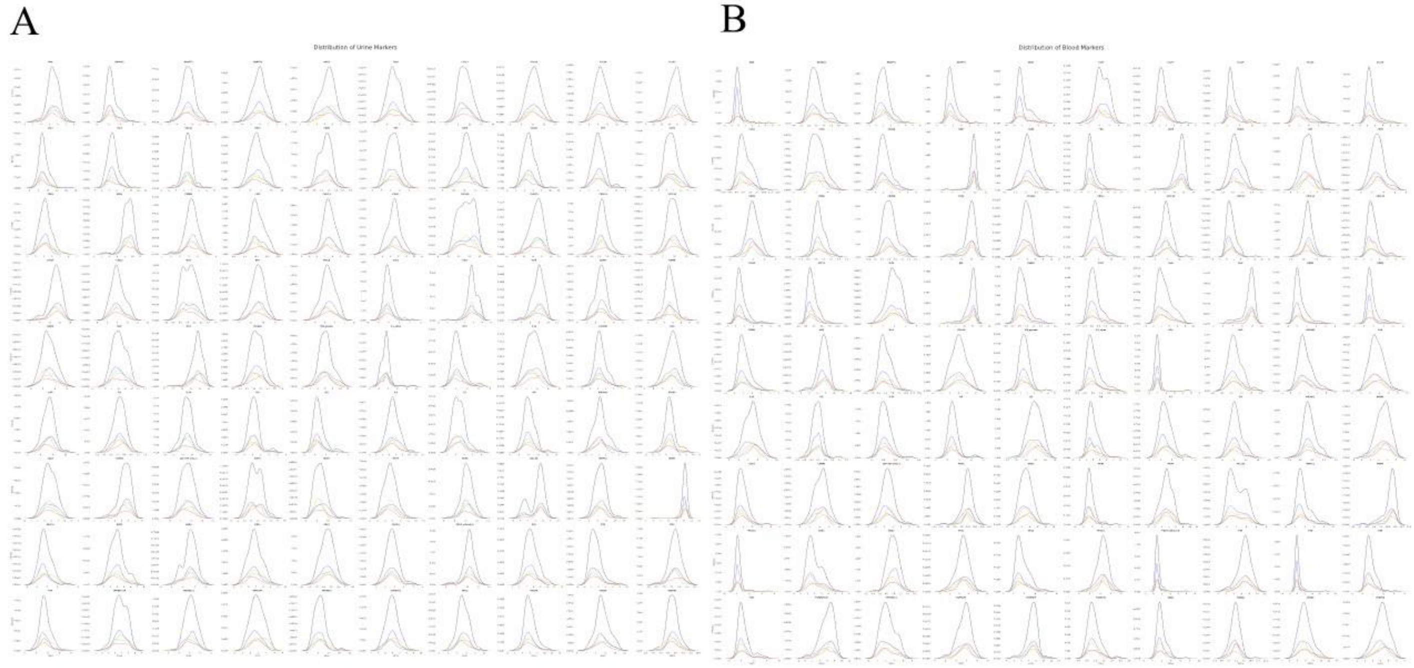

Supplementary Figure 4 Significant correlations of urine markers with CCI (A), LOS and LOS ICU (B) and APACHE at admission and 24 into hospital stay (C)

A

| | CCI |
|---|---|
| DCN | 0.552 |
| CAIX | 0.434 |
| PTN | 0.425 |
| TNFRSF12A | 0.398 |
| Gal-9 | 0.386 |
| IL33 | 0.378 |
| CX3CL1 | 0.375 |
| ADGRG1 | 0.367 |
| PGF | 0.360 |
| HGF | 0.353 |
| CSF-1 | 0.349 |
| CD27 | 0.344 |
| Gal-1 | 0.344 |
| CRTAM | 0.337 |
| TWEAK | 0.318 |
| IL15 | 0.315 |
| TNFRSF21 | 0.302 |
| HO-1 | 0.279 |
| CD40 | 0.277 |
| CD28 | 0.263 |
| CXCL12 | 0.252 |
| IL18 | 0.227 |
| NCR1 | 0.226 |
| LAMP3 | 0.226 |
| CD70 | 0.226 |
| CXCL13 | 0.214 |
| ICOSLG | 0.210 |
| CXCL9 | 0.194 |
| MIC-A/B | 0.190 |
| PDGF subunit B | 0.128 |
| PDCD1 | -0.007 |
| IFN-gamma | -0.213 |
| CCL17 | -0.218 |
| MCP-4 | -0.297 |
| IL5 | -0.298 |

B

| | LOS | LOS.ICU |
|---|---|---|
| MCP-3 | 0.367 | 0.361 |
| MUC-16 | 0.336 | 0.258 |
| CXCL13 | 0.299 | 0.277 |
| MCP-1 | 0.288 | 0.288 |
| CCL4 | 0.273 | 0.255 |
| IL5 | 0.261 | 0.295 |
| CCL23 | 0.236 | 0.224 |
| CCL20 | 0.235 | 0.205 |
| MCP-4 | 0.231 | 0.202 |
| PDCD1 | 0.217 | 0.197 |
| ANGPT2 | 0.196 | 0.202 |
| LAMP3 | 0.194 | 0.188 |
| IL8 | 0.189 | 0.193 |
| TNF | 0.189 | 0.162 |
| GZMH | 0.187 | 0.223 |
| NOS3 | 0.176 | 0.194 |
| LAP TGF-beta-1 | -0.159 | -0.220 |
| TWEAK | -0.235 | -0.229 |
| IL7 | -0.240 | -0.246 |
| CD244 | -0.247 | -0.273 |
| MIC-A/B | -0.268 | -0.292 |
| VEGFR-2 | -0.272 | -0.198 |
| PDGF subunit B | -0.279 | -0.267 |
| ANGPT1 | -0.290 | -0.319 |
| KIR3DL1 | -0.316 | -0.296 |
| CXCL5 | -0.320 | -0.341 |
| EGF | -0.381 | -0.409 |

Sample Size

| Urine | | | | Blood | | | |
|---|---|---|---|---|---|---|---|
| H1 | H2 | H3 | H4 | H1 | H2 | H3 | H4 |
| 17 | 23 | 37 | 25 | 22 | 26 | 41 | 25 |

C

| | APACHE1h | APACHE24h |
|---|---|---|
| TNFRSF12A | [illegible] | [illegible] |
| PGF | 0.565 | 0.507 |
| TNFRSF21 | 0.542 | 0.505 |
| CAIX | 0.516 | 0.441 |
| DCN | 0.502 | 0.441 |
| IL15 | 0.490 | 0.332 |
| CD27 | 0.473 | 0.424 |
| CD83 | 0.467 | 0.456 |
| CX3CL1 | 0.464 | 0.422 |
| Gal-9 | 0.452 | 0.427 |
| NCR1 | 0.430 | 0.437 |
| HGF | 0.429 | 0.375 |
| CCL23 | 0.415 | 0.370 |
| TNFRSF4 | 0.405 | 0.413 |
| CXCL13 | 0.400 | 0.318 |
| PD-L2 | 0.397 | 0.332 |
| CXCL9 | 0.397 | 0.380 |
| TNF | 0.382 | 0.342 |
| TNFRSF9 | 0.369 | 0.347 |
| Gal-1 | 0.369 | 0.387 |
| VEGFA | 0.369 | 0.359 |
| HO-1 | 0.361 | 0.342 |
| IL8 | 0.350 | 0.314 |
| LAMP3 | 0.347 | 0.370 |
| IL12RB1 | 0.343 | 0.343 |
| CD4 | 0.340 | 0.321 |
| NOS3 | 0.337 | 0.328 |
| MCP-1 | 0.337 | 0.284 |
| IL18 | 0.332 | 0.336 |
| MUC-16 | 0.330 | 0.290 |
| CD40 | 0.323 | 0.307 |
| TWEAK | 0.321 | 0.226 |
| CSF-1 | 0.320 | 0.252 |
| CCL4 | 0.316 | 0.250 |
| PD-L1 | 0.315 | 0.284 |
| MMP12 | 0.303 | 0.371 |
| PTN | 0.301 | 0.302 |
| KLRD1 | 0.299 | 0.272 |
| ARG1 | 0.298 | 0.242 |
| ADA | 0.281 | 0.216 |
| MCP-3 | 0.250 | 0.209 |
| ANGPT2 | 0.236 | 0.245 |
| MMP7 | 0.236 | 0.226 |
| CXCL10 | 0.235 | 0.176 |
| CCL3 | 0.234 | 0.249 |
| IL10 | 0.232 | 0.273 |
| LAG3 | 0.230 | 0.217 |
| CXCL12 | 0.224 | 0.195 |
| IL6 | 0.218 | 0.281 |
| ICOSLG | 0.215 | 0.183 |
| PDCD1 | 0.200 | 0.190 |
| IL2 | 0.191 | 0.078 |
| ADGRG1 | 0.185 | 0.343 |
| CCL20 | 0.165 | 0.188 |
| CCL19 | 0.096 | 0.200 |
| IFN-gamma | -0.150 | -0.191 |
| EGF | -0.208 | -0.172 |
| CXCL5 | [illegible] | -0.221 |

Supplemental Figure 4

Supplemental Figure 5. Markers significant for demise (A), or ICU admission (B) when AKI and CKD patient were excluded.

A

**Disposition**
Alive Compared To Dead
(AKI & CKD Excluded)

| | Significance | | Mean Difference | |
|---|---|---|---|---|
| | Urine | Blood | Urine | Blood |
| ADGRG1 | 0.003 | 0.436 | -0.692 | -0.119 |
| ICOSLG | 0.001 | 0.559 | -0.281 | -0.743 |
| IL7 | 0.001 | 0.764 | -0.599 | 0.094 |
| PTN | 0.007 | 0.811 | -0.972 | -0.030 |
| ANGPT1 | 0.044 | 0.007 | -1.093 | -0.949 |
| CCL17 | 0.016 | 0.462 | -1.321 | -0.151 |
| MCP-2 | 0.013 | 0.035 | -1.102 | -1.274 |
| TWEAK | 0.011 | 0.098 | -1.414 | -1.416 |
| CD244 | 0.139 | 0.000 | -1.025 | -1.011 |
| CD5 | 0.425 | 0.007 | -0.511 | -0.930 |
| CRTAM | 0.376 | 0.029 | -0.311 | -0.540 |
| CXCL9 | 0.539 | 0.007 | -0.221 | 1.030 |
| FASLG | 0.713 | 0.000 | -0.146 | -0.420 |
| GZMB | 0.697 | 0.010 | 0.142 | -0.651 |
| GZMH | 0.147 | 0.001 | -1.159 | -0.555 |
| IFN-gamma | 0.177 | 0.042 | 0.435 | -0.442 |
| IL-1 alpha | 0.845 | 0.000 | 0.039 | -2.245 |
| IL33 | 0.308 | 0.004 | -0.233 | -0.174 |
| KIR3DL1 | 0.567 | 0.000 | 0.760 | -0.267 |
| LAMP3 | 0.052 | 0.000 | 0.795 | -1.323 |
| MMP12 | 0.300 | 0.014 | 0.814 | -0.637 |
| PDCD1 | 0.528 | 0.040 | -0.303 | -1.185 |
| TNFSF14 | 0.472 | 0.010 | -0.796 | -0.163 |
| ANGPT2 | 0.021 | 0.317 | 0.905 | 0.891 |
| CD8A | 0.030 | 0.585 | 1.343 | -0.054 |
| MIC-A/B | 0.020 | 0.813 | 0.957 | 0.310 |
| NCR1 | 0.011 | 0.059 | 0.560 | -0.691 |
| NOS3 | 0.031 | 0.145 | 0.485 | -0.099 |
| TNFRSF4 | 0.021 | 0.962 | 0.617 | 0.040 |
| CCL20 | 0.004 | 0.448 | 1.551 | -0.490 |
| CCL23 | 0.008 | 0.371 | 0.577 | 0.806 |
| CCL3 | 0.000 | 0.328 | 0.639 | 2.154 |
| CD27 | 0.000 | 0.409 | 0.446 | -1.642 |
| IL8 | 0.000 | 0.936 | 0.836 | 0.140 |
| LAG3 | 0.009 | 0.006 | 0.870 | -0.176 |
| MCP-1 | 0.000 | 0.650 | 0.706 | -0.934 |
| MMP7 | 0.000 | 0.683 | 0.483 | -1.008 |
| MUC-16 | 0.000 | 0.266 | 1.099 | -0.200 |
| PGF | 0.008 | 0.917 | 0.433 | -0.154 |
| TNFRSF12A | 0.008 | 0.612 | 0.735 | 0.765 |

B

**Disposition**
ICU Compared To No ICU
(AKI & CKD Excluded)

| | Significance | | Mean Difference | |
|---|---|---|---|---|
| | Urine | Blood | Urine | Blood |
| CXCL13 | 0.006 | 0.110 | -0.746 | -0.357 |
| Gal-9 | 0.001 | 0.997 | -0.680 | 0.002 |
| HO-1 | 0.000 | 0.282 | -0.634 | 0.629 |
| IL18 | 0.000 | 0.667 | -0.942 | 0.166 |
| LAMP3 | 0.000 | 0.898 | -1.028 | 0.049 |
| MCP-3 | 0.003 | 0.057 | -1.265 | -0.379 |
| PD-L1 | 0.003 | 0.380 | -0.722 | -0.318 |
| ADA | 0.014 | 0.122 | -0.603 | 0.201 |
| CD27 | 0.013 | 0.980 | -0.401 | 0.006 |
| CD70 | 0.017 | 0.498 | -0.396 | -0.210 |
| CX3CL1 | 0.010 | 0.812 | -0.559 | -0.087 |
| IL15 | 0.019 | 0.136 | -0.417 | -0.574 |
| MCP-1 | 0.011 | 0.180 | -0.609 | -0.805 |
| MUC-16 | 0.020 | 0.361 | -0.796 | 0.139 |
| NOS3 | 0.042 | 0.758 | -0.623 | 0.035 |
| TNFRSF12A | 0.017 | 0.003 | -0.647 | -1.477 |
| ANGPT2 | 0.499 | 0.021 | -0.172 | -0.290 |
| CAIX | 0.054 | 0.015 | -0.510 | -0.757 |
| CCL19 | 0.473 | 0.019 | 0.219 | -0.709 |
| CXCL1 | 0.785 | 0.046 | -0.070 | 1.340 |
| Gal-1 | 0.052 | 0.022 | -0.403 | -0.840 |
| IL-1 alpha | 0.228 | 0.011 | 0.356 | 1.737 |
| TNFRSF21 | 0.130 | 0.009 | -0.227 | -0.905 |
| TNFSF14 | 0.336 | 0.029 | -0.241 | 0.256 |
| ICOSLG | 0.030 | 0.353 | 0.328 | 0.430 |
| IL12 | 0.011 | 0.441 | 0.960 | 0.063 |

**Sample Size**

| Urine | | | | Blood | | | |
|---|---|---|---|---|---|---|---|
| H1 | H2 | H3 | H4 | H1 | H2 | H3 | H4 |
| 17 | 23 | 37 | 25 | 22 | 26 | 41 | 25 |

Supplemental Figure 5

Supplemental Figure 6. Differences in the values of MCP-1 (A) and MCP-3 (B) in patients who develop AKI vs not over admission times.

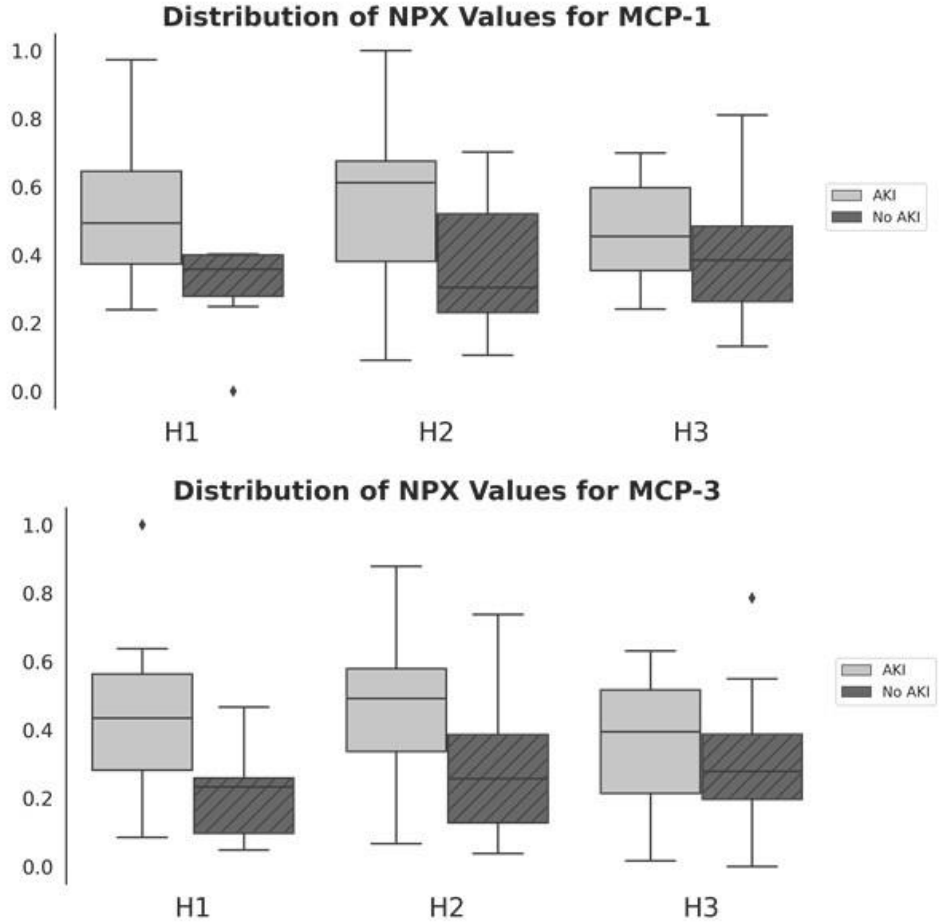


Sample Size

| Urine | | | | Blood | | | |
|---|---|---|---|---|---|---|---|
| H1 | H2 | H3 | H4 | H1 | H2 | H3 | H4 |
| 17 | 23 | 37 | 25 | 22 | 26 | 41 | 25 |
